# Addressing gaps in surgical skills training by means of low-cost simulation at Muhimbili University in Tanzania

**DOI:** 10.1186/1478-4491-7-64

**Published:** 2009-07-27

**Authors:** Stephanie Taché, Naboth Mbembati, Nell Marshall, Frank Tendick, Charles Mkony, Patricia O'Sullivan

**Affiliations:** 1 Global Health Sciences, University of California San Francisco, San Francisco, California, USA; 2 School of Medicine, Muhimbili University of Health and Allied Sciences, Dar es Salaam, Tanzania; 3 Department of Health Services, School of Public Health, University of California Los Angeles, Los Angeles, California, USA

## Abstract

**Background:**

Providing basic surgical and emergency care in rural settings is essential, particularly in Tanzania, where the mortality burden addressable by emergency and surgical interventions has been estimated at 40%. However, the shortages of teaching faculty and insufficient learning resources have hampered the traditionally intensive surgical training apprenticeships. The Muhimbili University of Health and Allied Sciences consequently has experienced suboptimal preparation for graduates practising surgery in the field and a drop in medical graduates willing to become surgeons. To address the decline in circumstances, the first step was to enhance technical skills in general surgery and emergency procedures for senior medical students by designing and implementing a surgical skills practicum using locally developed simulation models.

**Methods:**

A two-day training course in nine different emergency procedures and surgical skills based on the Canadian Network for International Surgery curriculum was developed. Simulation models for the surgical skills were created with locally available materials. The curriculum was pilot-tested with a cohort of 60 senior medical students who had completed their surgery rotation at Muhimbili University. Two measures were used to evaluate surgical skill performance: Objective Structured Clinical Examinations and surveys of self-perceived performance administered pre- and post-training.

**Results:**

Thirty-six students participated in the study. Prior to the training, no student was able to correctly perform a surgical hand tie, only one student was able to correctly perform adult intubation and three students were able to correctly scrub, gown and glove. Performance improved after training, demonstrated by Objective Structured Clinical Examination scores that rose from 6/30 to 15/30. Students perceived great benefit from practical skills training. The cost of the training using low-tech simulation was four United States dollars per student.

**Conclusion:**

Simulation is valued to gain experience in practising surgical skills prior to working with patients. In the context of resource-limited settings, an additional benefit is that of learning skills not otherwise obtainable. Further testing of this approach will determine its applicability to other resource-limited settings seeking to develop skill-based surgical and emergency procedure apprenticeships. Additionally, skill sustainability and readiness for actual surgical and emergency experiences need to be assessed.

## Background

### Surgery as an essential service

Injury is a growing epidemic in East Africa and large numbers of injured people are at risk of death and lifelong disability [1]. Surgery has a major role to play in public health in the prevention of death and disability in addition to treating injuries, including obstetrical emergencies and a wide range of emergency abdominal and non-abdominal conditions [2-4].

The second edition of *Disease control priorities in developing countries *(DCP2) has brought attention to the role of surgery as a public health strategy [5]. The DCP2 estimated that 11% of all disability-adjusted life years (DALYs) are from conditions likely to require surgery. Furthermore, the report demonstrated that surgical services provided in low-cost district hospitals in resource-constrained countries are highly cost-effective. Yet a large amount of unmet need persists, due to untrained health workforce and inadequate surgical infrastructure in rural areas. One study in Tanzania used "met need" and "case fatality" to estimate the contribution of surgical access in two regions with a combined population of five million. In both regions, the "met need" amounted to 30%. The DCP2 suggests there is an urgent need to expand the number of health facilities with the ability to provide access and services to emergency obstetrical surgery [5].

One of the primary obstacles to expanding surgical services is the relatively few trained surgeons to care for more than 200 million people in the eight-country region of East Africa. The limited numbers of medical graduates in Tanzania are preferentially posted to District Medical Officer (DMO) positions in rural areas, where they are expected to perform essential surgical and emergency obstetric care. Improving the quality of their training is therefore a means of improving patient outcomes.

### Surgical training in Tanzania

The Muhimbili University of Health and Allied Sciences (MUHAS) trains the greatest number of medical students in Tanzania. Several challenges have constrained MUHAS' ability to properly train medical students to serve these roles.

The medical school has experienced an explosion in the number of students enrolled in the past five years, without a proportional increase in teaching resources or faculty. In surgery, the faculty-to-student ratio has decreased from 1:2 to 1:10, with less individual teaching time – an essential part of surgical skills apprenticeship [6]. The teaching space in surgical theatres at the Muhimbili National Hospital, designed to accommodate a maximum of five medical students, is inadequate for the 60 medical students carrying out their surgical clerkship at any given time. The surgical curriculum has no dedicated time to supervise and teach practical skills outside of operating theatre cases.

Limited space and decreases in surgical case-load due to major hospital rehabilitation work has resulted in students' being able to attend only a handful of surgeries during their eight-week surgical clerkship. Lack of preparation at the medical student level extends into internship, when interns believe they are ill-equipped to perform the skills necessary for surgical specialization, which affects their career choices [7].

### The role of simulation in surgical training

One way to improve surgical skills training has been to transfer parts of the surgical apprenticeship to laboratory settings, by means of simulation models. Such surgical training, for example, in tying, suturing and instrument handling, has been shown to reduce the failure rate after formal training [8]. Such skills laboratories have been found to be the type of training rated highest by students [9]. Early exposure can also improve student attitudes towards surgery as a career [10,11].

Although there is an increasing amount of literature on simulated surgical training, we found no published reports on surgical skills training for medical students in Africa. The Canadian Network for International Surgery (CNIS) has had the greatest amount of experience in surgical training with simulation in Africa, with 5000 primary care physicians in Ethiopia, Malawi, Mozambique, Tanzania, and Uganda having benefited from training through the essential surgical skills programme [12].

In order to address the gaps in surgical training of medical students at MUHAS, we created, implemented and evaluated a low-cost surgical skills curriculum using limited technology for senior medical students carrying out their surgery clerkship. The goal of the training was to teach medical students the fundamental skills and knowledge required to assist with surgical procedures and feel comfortable in the operating room in a context with limited resources. The purpose of this article is to describe the creation and evaluation of a surgical skills training course for senior medical students developed and implemented by the MUHAS Department of Surgery.

## Methods

### Curriculum development

To guide the development of the curriculum, we conducted a needs assessment of surgical faculty to determine the gaps in surgical and emergency procedures training for the surgery clerkship between what is offered and what is needed. Seven members of the surgical teaching faculty in the MUHAS Departments of Surgery, Orthopaedics, Obstetrics and Anaesthesiology were interviewed individually to get their feedback on the skills actually taught and those they believed students were missing. The Canadian Network for International Surgery (CNIS) surgical training curriculum used to train interns, registrars and primary care consultants at the Muhimbili National Hospital was also reviewed as part of the needs assessment. Based on these findings, the CNIS curriculum was adapted with the overall goal of improving essential surgical and emergency skills. The two-day curriculum covered nine skills in the fundamentals of assisting in the operation room (OR), general surgical skills, anaesthesia skills and emergency obstetrical skills (Table 1).

**Table 1:** List of skills taught, with length of time and materials used for the surgical skills training course

**Surgical skills**	**Objectives**	**Time in curriculum**	**Models used**
Scrubbing, gowning & gloving (OSCE)	To demonstrate proper techniques for surgical scrubbing and donning of surgical gowns and gloves while maintaining sterile field	55 minutes	Cloth theatre gowns, plastic gloves

Patient preparation	To demonstrate proper technique for patient skin sterilization to prepare field prior to surgery	55 minutes	Wood bench draped with plastic with abdominal landmarks (Figure 2)

Surgical knot tying (OSCE)	To demonstrate proper knot tying techniques and their indications	110 minutes	Ethicon knot tying models (Figure 3)

Venous cutdown (OSCE)	To learn indications for venous cutdown; identify the anatomical location of long saphenous vein; perform a venesection using simulated leg	55 minutes	Plastic tube placed on arm stabilizer covered with foam & vinyl

Adult intubation (OSCE)	To demonstrate technique for adult intubation: visualize laryngeal cords, insert endotracheal tube and properly ventilate using an ambu bag	55 minutes	Adult intubation mannequin ("Airway Larry")

Chest tube insertion	To state the indications for chest tube insertion and demonstrate the technique for chest tube insertion	55 minutes	Teaching skeleton rib cage draped with foam & vinyl (Figure 4)

Laparotomy (OSCE)	To open anterior abdominal wall using midline incision and close abdomen in layers using mattress sutures	55 minutes	Wood frame with foam, clear & colored vinyl (Figure 5)

Small bowel repair	To understand the principles of bowel repair and perform repair of perforated bowel	55 minutes	Inner tubes from bicycle tires (Figure 6)

Vacuum-assisted vaginal delivery	To know the indications for vacuum-assisted delivery; demonstrate positioning of suction cup and proper guidance of the delivery	55 minutes	Obstetrical mannequin

### Curriculum Implementation

**Figure 1:**
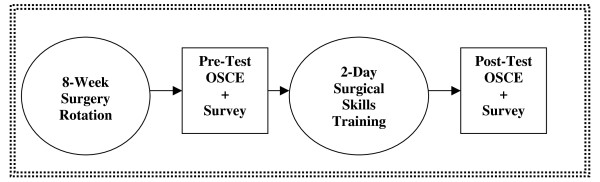
**Surgical skills study design**.

The planned curriculum included five one-hour training sessions over a two-day period held in the MUHAS Department of Anatomy Laboratory. Thirty-six of the 60 eligible students (60%) agreed to participate. They were divided into two groups of 18 for convenience purposes and completed the two-day training course after completing their mandatory eight-week surgery rotation (Figure 1). Students were arranged three to four per laboratory table, with eight surgical faculty, surgical technicians and surgical nurses providing instruction at each session (Table 2). Thus we had a student-faculty ratio of 3:1 to ensure the optimum recommended by current research [13]. For each of the five skills being taught each day, faculty members gave a five-minute introduction followed by 55 minutes of hands-on instruction (Table 1). Students rotated through five stations each day. The knot-tying station was repeated on the second day of the training, as it was deemed an essential skill for students to master.

**Table 2:** Budget and work-hours for surgical skills training

**Budget & personnel**				
Supplies				

Item	Quantity	Cost/Item (USD)	Total (USD)	

Local consumable surgical supplies	37	15	555	

Certified instructor work-days	36	40	1440	

General course support work-days	12	40	480	

		Total	2475	

Work-hours				

Person	Number	Hrs/day	No. days	Total

Surgical faculty	4	7	4	112 h

Surgical technicians	4	7	5	140 h

Training coordinator	2	2	6	24 h

Administrator	1	2	3	6 h

Student organizer	2	2	2	8 h

			Total	290 h

To teach students operating room etiquette with proper scrubbing, gowning, gloving and maintaining a sterile field, we worked with two operating-theatre nurse teachers. For the knot-tying and suturing instruction, we used the Ethicon Knot Tying Manual [14]. For the surgical abdominal incision, bowel anastomosis, vacuum extraction delivery and chest tube insertion stations, we worked with surgeons who had taught these skills in prior CNIS training courses at MUHAS.

**Figure 2:**
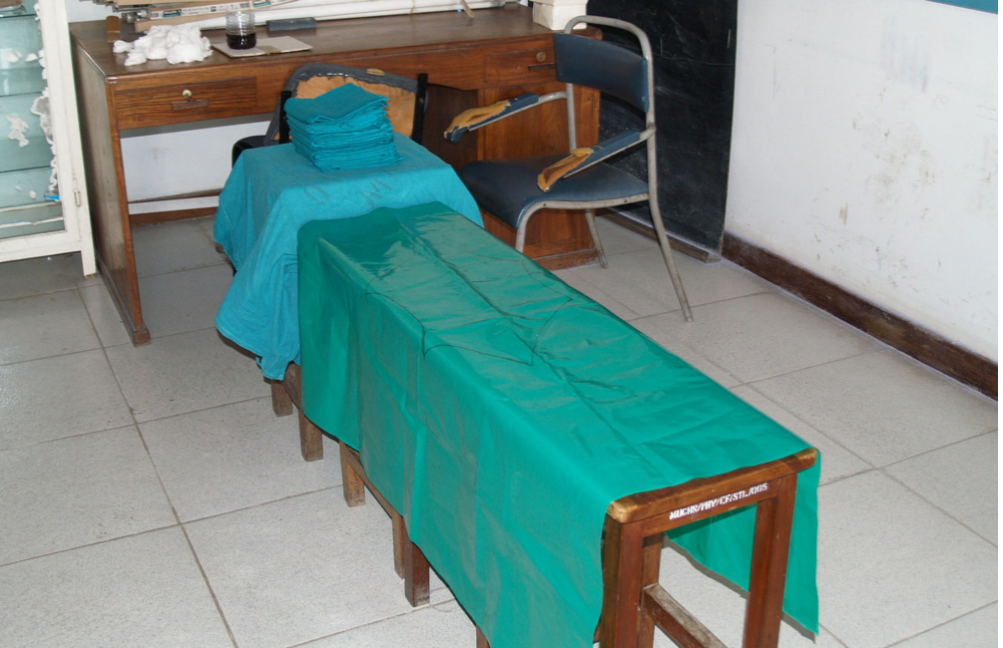
**Picture of bench draped with polythene sheet for a patient preparation station**.

Models used for the training course are listed in Table 1. The patient-preparation station consisted of a wooden bench draped with plastic on which abdominal anatomical landmarks were drawn. A colored solution was used in lieu of iodine for cleaning (Figure 2).

**Figure 3:**
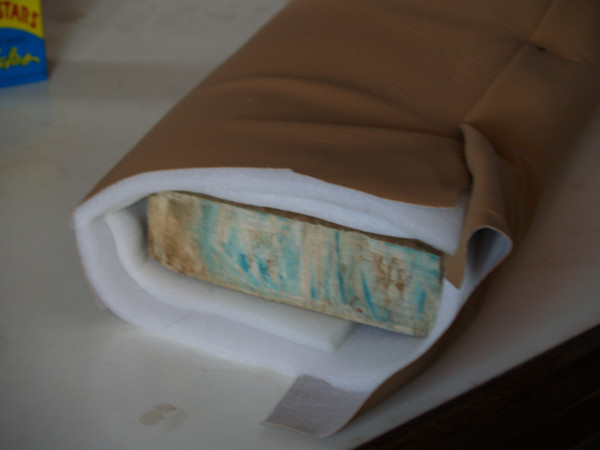
**Picture of foam suturing boards for knot-tying and suturing skills station**.

For the tying and suturing stations, we used coloured ropes and several types of sutures (2-0 vicryl, 2-0 prolene and 2-0 silk). Ethicon boards were used to practise knot tying; handcrafted procedures boards were used to practice suturing (Figure 3).

The venous cutdown model consisted of a firm plastic tube placed on an arm stabilizer covered with foam and opaque vinyl. Students were to identify the anatomical location of the long saphenous vein on themselves, then perform a venesection and insert the intravenous cannula into the vein of the model and secure the cannula in place.

**Figure 4:**
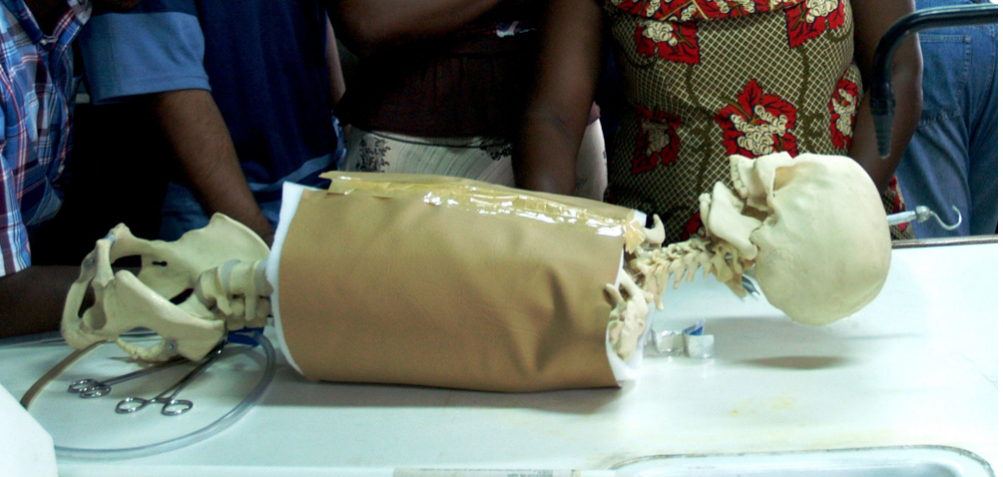
**Picture of skeleton model for chest tube insertion skills station**.

The intubation station was carried out using adult intubation mannequins ("Airway Larry," Nasco Health Care) and adult laryngoscopes. The chest model used to teach chest tube insertion consisted of a teaching-skeleton rib cage draped with layers of foam and opaque vinyl that were sewn shut (Figure 4). Students were to review anatomy on a volunteer male student, isolating the fifth rib on the anterior axillary line, insert the chest tube in the mannequin, secure the tube in place with suture, and close the chest tube opening with a suture upon removal of the tube.

**Figure 5:**
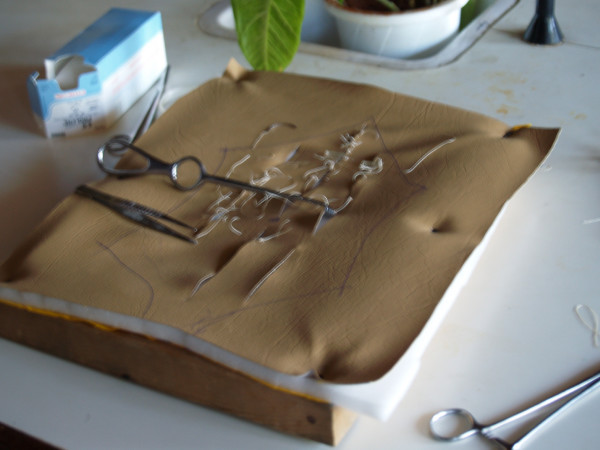
**Picture of model used for laparotomy skills station**.

For the laparotomy station, an abdominal frame consisting of a square wood frame covered with three layers of foam, clear vinyl and colored vinyl, respectively, was used (Figure 5). Students practised how to open the abdomen by midline incision and close the abdomen in layers with mattress and tension (retention) sutures.

**Figure 6:**
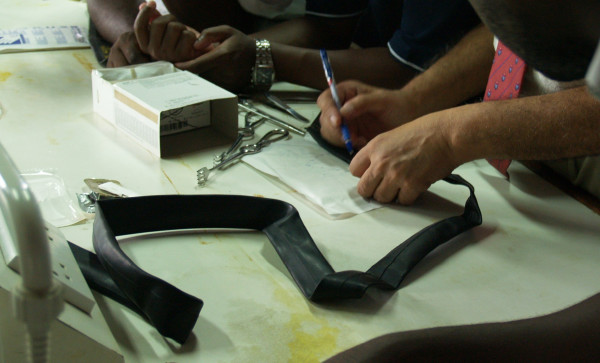
**Picture of inner tube models for bowel anastomosis station**.

For the small-bowel repair station, we used inner tubes from bicycle tires to mimic small bowel (Figure 6). Students learned how to make a perforation and repair the perforation without narrowing the bowel lumen.

For the vacuum-assisted vaginal delivery, we used an obstetrical mannequin on which students practised appropriate placement of the vacuum on the fetal head and developed proper delivery traction with the vacuum.

The total budget for these materials was USD 555, outlined in Table 2. The obstetrical mannequin and one of the adult intubation mannequins, as well as the surgical instruments, belonged to MUHAS. Additionally, sutures and an extra intubation mannequin were donated by the University of California San Francisco (UCSF) Global Health Sciences.

Development and execution of the training required a total of 290 work-hours (Table 2). The execution of the surgical skills training required a total of 252 hours of faculty teaching time. The surgical faculty contributed an additional eight hours in curriculum development meetings. The chairman of the Department of Surgery at Muhimbili University coordinated and oversaw the entire process. The ultimate goal of the training was to provide opportunities for medical students to have hands-on apprenticeship of surgical skills outside the operating theatre.

### Evaluation

The study design was a pre/post evaluation of a training intervention in a cohort of Tanzanian senior medical students carrying out their surgery clerkship at Muhimbili University (Figure 1). For ethical reasons, all 60 students enrolled in the surgical clerkship were offered the option to participate in the training. The inclusion of a control group from this same cohort would have entailed repeating the surgical skills training workshop for this group after study completion for ethical reasons. However, we did not have the human resource capacity to carry out this second training; for this reason, a pre/post evaluation of the training intervention was agreed upon.

Students were evaluated by means of two methods – a questionnaire survey and an Observed Structured Clinical Examination (OSCE) – before and after participating in the two-day surgical skills training course. The surgical performance evaluation was used only to measure effectiveness of the training, and was not incorporated into students' final grades for their surgery clerkship. This study was performed with the approval of the Muhimbili Ethics Committee and the UCSF Institutional Review Board.

Students completed pre- and post-training questionnaires to measure self-perceived confidence in performing select surgical procedures. After training, they also provided their overall satisfaction with the training. Quantitative data gathered also included demographic data, information on previous surgical experience, and whether students envisaged surgery to be part of their future clinical practice and career. The surveys rated student confidence in each of the skills listed in Table 1. Students rated their confidence on a five-point Likert scale, or (1) "Very unconfident" to (5) "Very confident". The post-training survey also included qualitative questions on how the training changed students' perception of practising surgery as well as recommendations for improvement of the training.

The OSCEs that measured baseline surgical skills performance and acquisition of skills as a result of the training focused on four of nine skills taught during the course that were identified as most relevant and measurable (Table 1). Because the same teaching faculty members evaluated the OSCEs and were not blinded as to whether the students were at the pre-course or post-course stage, a strict grading system for the skills being observed was developed and agreed upon prior to the exams.

To minimize biased ascertainment of the outcome, faculty members were instructed to adhere to the same grading system. Four to six critical steps were identified for each skill observed, and were evaluated as performed correctly or incorrectly by the observing faculty, for a total of 30 possible points over the five rated skills. For example, for the scrubbing, gowning and gloving station, five steps were scored: washing in the fingers to elbow fashion; drip drying with hands-up technique; drying hands correctly, making sure not to repeat same site; donning gloves without touching fingerstalls; and doing up the gown properly without breaking the sterile field.

We calculated descriptive statistics for the items on the survey including percentages, means and standard deviations. We used matched paired t-tests to compare performance pre- to post for those students participating in the surgical skills study. We also calculated effect sizes to identify the magnitude of the effect of the intervention independent of sample size. Effect sizes exceeding 0.8 are considered large [15].

While completing the post-test survey, students were also asked to respond to two open-ended questions: "How has this training changed your perceptions about practising surgery?" and "How would you improve this training?" These written, qualitative responses were entered into an Excel database and reviewed independently by two researchers to identify general themes. The qualitative data were then coded and organized within these themes, based on their frequency.

## Results

Thirty-eight of 60 eligible fifth-year medical students who had completed their eight-week surgery clerkship consented to participate in the study. We could match pre- and post data for 36 of the participants. Twenty-four of the participants were male and 12 were female. Their average age was 26 years (range 22 to 34). All participants anticipated practising general surgery after completing medical school. Seventy seven percent of participants reported they were likely or very likely to practise general surgery, while 33% stated they were somewhat likely to practise surgery.

Students reported a low number of times scrubbing into the OR, suturing or knot-tying prior to the training course. Among participating students, the average number of times entering the operating theatre was seven, with 84% of the students estimating they had observed a surgical procedure 10 times or less; 70% of the students reported scrubbing and gowning for a procedure one to five times. Twenty-one percent of the students had no prior opportunity to practice suturing (an average of 1.4 times/student), while 46% had not performed surgical knot-tying (an average of 2.4 times/student) during their surgery rotation (Table 3).

**Table 3:** Surgery rotation experience for students participating in surgical skills training

Frequency in number of times	**Number of students reporting having engaged in specified activity during their surgery rotation**			
	
	Observed a surgery in operating room	Scrubbed & gowned for a surgical case	Opportunity to suture	Practise surgical knot-tying
0	0/37	0/37	8/37	17/37

1 to 5	15/37	26/37	27/37	19/37

6 to 10	16/37	10/37	1/37	1/37

>10	6/37	1/37	1/37	0/37

Students scored an average of 6.3 out of 30 points (SD 3.2) on the pre-training OSCE. No student was able to correctly carry out the steps for a surgical hand tie or an instrument tie. Only one student was able to perform the correct steps for adult intubation; three out of 36 students were able to correctly scrub, gown and glove (Table 4).

**Table 4:** Mean score improvement in surgical skills OSCE evaluation

	**Pre-OSCE**	**Post-OSCE**	**Mean Difference**	**SD**	**p**	**Effect size**
Scrubbing, gowning & gloving	2.74	4.51	1.77	1.14	<.001	1.55

Hand knot-tying	0.43	5.29	4.86	1.15	<.001	4.23

Surgical knot-tying	0.03	3.41	3.39	1.06	<.001	3.20

Venous cut down	1.57	4.71	3.14	1.44	<.001	2.18

Laparotomy	0.71	4.13	3.41	1.28	<.001	2.66

Adult intubation	1.41	3.94	2.53	1.49	<.001	1.70

Performance on the post-training OSCE demonstrated a fourfold improvement in skill. Scores increased by 19.4 points (SD 4.0). Improvements were most significant for knot-tying skills, with a 3.39-point mean increase in skill for the instrument ties and a 4.86-point mean increase for the hand ties (Table 4). Eighty-six percent of students (20/36) were able to correctly perform an instrument tie and 63% (23/36) were able to perform a surgical hand tie after the surgical skills training; 81% of the students (31/36) correctly performed the steps for adult intubation. Gains in surgical skills after the training were lowest for venesection, with only 27% (10/36) of the students performing all the correct steps (mean score 3.8/5), and laparotomy, with 33% (12/36) correct performance (mean score 4.0/5) (Table 4).

Eighty-nine percent of the respondents strongly agreed that the training was a valuable use of their break time, believed it would help them provide better patient care and would recommend the training to other medical students.

The training changed their perception about practising surgery. Of the 37 students who completed the surveys, 28 contributed qualitative feedback. Students reported an increase in willingness and preparedness to carry out surgery after the training. Comments from participants included: "I am starting to change my mind for practicing surgery in the future while before I was thinking of internal medicine". Post-training survey results reflect this phenomenon where students commented: "Despite not having considered surgery as an option in what I wanted to specialize at the beginning, I have realized it much more interesting than it seemed and I can learn, practice and be good at it."

Improvement themes related to wanting longer duration of training and broadening of the skills covered to include procedures such as caesarean section. There was overall satisfaction with the manner in which the skills were taught (hands-on apprenticeship with oral guidance) and the individual attention each student received to learn the skills. However, the two days of training did not significantly change self-perceived confidence levels of surgical skills or participants' likelihood to practise general surgery after medical school.

## Discussion

### Surgical training at MUHAS

Although the acquisition and mastery of basic surgical procedures, trauma management and emergency obstetrical skills are essential for medical graduates, the quality of surgical training at Muhimbili University has declined in recent years. This study highlights how surgical training has been affected at the medical student level. The decline in surgical training is reflected in the low mastery of practical skills in the pre-training OSCE. While students may have the opportunity to enter the operating theatre, there is little opportunity to practise surgical skills in the current training context.

Reasons why students have limited opportunity to assist in surgeries are most likely related to the expanding number of students needing to be accommodated during surgery clerkships. Thus, even though five students are allowed to scrub into an operation at any given time, it is impractical for all of them to assist on the case. The introduction of simulation in teaching surgical skills is a feasible solution to redressing surgical performance in this group. The need to emphasize surgical skills mastery at the medical student level is important for the purpose of improving quality of patient care, but also to increase the odds of students' pursuing surgical practice or surgery as a career.

### Training effectiveness

The use of low-fidelity and low-cost simulation has been demonstrated to be an effective alternative to high-fidelity computer simulation [16-18]. This was confirmed in our study with measurable improvements in performance before and after exposure to surgical skills training intervention, as demonstrated by the increase in OSCE scores. Our effect sizes were large, indicating that the significant improvement was most likely related to the intervention and not sample size.

Although we believe the increase in performance was a result of increased skill performance, there are other potential explanations for this increase independent of actual performance. One possibility is that performance improvement was a result of rater bias or expectations from the non-blinded faculty raters. We believe that the OSCE rating system we developed focusing on the grading of discrete skills mitigates the likelihood of rater bias.

Other explanations for performance improvement not related to the two-day course include short-term Hawthorne effects (the natural improvements that occur from receiving attention), the effect of repeated observation and the effect of repeated testing. We believe that the magnitude of the improvement, as indicated by the large effect sizes, combined with the complexity of skills being taught, cannot be a result solely of these factors. However, it is possible that all these factors influence the magnitude of the increase in performance seen in our cohort of participants.

The confidence levels reported by students were not significantly changed by the exposure. One reason for this may be that the duration of the training was not long enough for students to boost their self-confidence in performance.

This study capitalized on existing expertise in teaching surgical skills at MUHAS, a result of CNIS annual training-of-trainers workshops in surgical skills since 2003. Eight of the 19 teaching faculty members in the MUHAS Department of Surgery trained in this way took part in the study. Beyond the monetary resources to build such a centre, the presence and availability of teaching faculty versed in pedagogical methods for surgical skills are essential to the effectiveness of the training.

### Cost of training by means of simulation

We used low-technology models with locally available supplies to carry out the surgical skills training. Except for the Ethicon boards, the adult intubation models and obstetric mannequins, all models were locally produced at minimal cost. As there was an outbreak of West Nile virus at the time we performed the training, animal models were replaced by man-made models of wood, foam, rubber and vinyl. This eliminated many of the procurement, storage and disposal complexities associated with prior training courses conducted at MUHAS. One of the unintended consequences of this event was a considerably reduced materials cost of USD 15 per student (Table 2). Although some may question the fidelity of models made of wood, foam and vinyl to actual surgical cases, studies have shown that even low-fidelity simulation can improve skill levels [16,17].

### Impact on career choices

Irrespective of whether medical graduates choose surgery as a final career path, having proficiency in essential surgical and emergency obstetrical skills is necessary for Tanzanian physicians. This is due to the structure of medical training in Tanzania, where medical graduates are expected to practise in rural settings for one to three years after completing internship.

MUHAS has a strong culture of public service, and there is an underlying expectation that medical graduates will adopt rural postings upon completing medical school. This expectation is reflected in our study, where participants reported a high likelihood that they would be required to perform surgery in the future on both the pre- and post-training surveys. Thus, students were able to gain a basic level of mastery and begin entertaining the possibility of practising surgery as a career choice.

In addition to the implications for quality of patient care, surgical training has implications for the future production of surgeons in Tanzania. Although no data exist to determine what proportion of MUHAS medical graduates actually end up staffing district hospitals and working in rural areas, a study in 2003 to determine employment patterns of MUHAS graduates found that 76% of medical graduates eventually took advantage of opportunities for additional training [19]. In view of this high proportion of graduates going on to receive additional training, determining the extent to which additional skills training in medical school affects their selection of surgery as a career choice for postgraduate training is important for the future production of surgeons.

Feelings of inadequacy ultimately affect career choices: students who are plagued with preoccupations of incompetence about their skill levels do not end up in a field they feel they cannot master. Several students described surgery as something unattainable for them because the skills were too difficult to acquire. When the surgical process was demystified, students were able to gain a basic level of mastery and begin entertaining the possibility of practising surgery as a career choice.

## Conclusion

The use of simulation in teaching surgical skills fills an essential gap for which there is no substitute. Surgical skills learning facilities provide an educational environment in which protected time can be devoted to the development of a wide range of skills. Although these facilities are not intended to replace the learning derived from real clinical experience, they do enable the learners to establish a foundation in a range of skills that can subsequently be honed and made more substantial through experiential learning in surgical practice.

One limitation of this study is the lack of longitudinal data to demonstrate whether these skills were retained over time. Further study is required to measure the longer-term impact of such training with regard to skill retention and performance.

In order to redress the decline in surgical apprenticeship highlighted by this study, MUHAS, in association with CNIS and UCSF, will build a surgical skills laboratory on the main campus. Teaching of essential surgical procedures will therefore be integrated into the MUHAS curriculum in the near future so that students may learn skills that are otherwise not attainable. Plans to measure skill sustainability and readiness for actual surgical and emergency experiences will be imbedded into this programme.

## List of abbreviations

CNIS: Canadian Network for International Surgery; DALY: disability-adjusted life year; DCP: disease control priorities; DMO: District Medical Officer; MUHAS: Muhimbili University of Health and Allied Sciences; OR: operation room (or OT: operating theatre); OSCE: Observed Structured Clinical Examination; UCSF: University of California San Francisco.

## Competing interests

The authors declare that they have no competing interests.

## Authors' contributions

NB and ST contributed equally to the development of this manuscript and share first co-authorship. Additionally, NB participated in the needs assessment and curriculum design and coordinated the entire study; ST contributed to the study design and data collection; NM carried out data analysis and assisted with logistics and procurement of equipment and drafting of the manuscript; FT contributed to the study design and manuscript writing; CM provided writing assistance; PO assisted with data analysis and manuscript writing. All authors read and approved the final manuscript.

## References

[B1] World Health Organization (2006). Working Together for Health: The World Health Report 2006.

[B2] Nordberg E (1994). Injuries in Africa: A review. East African Medical Journal.

[B3] World Health Organization W Surgical and emergency obstetrical care at first referral level. Aide-Memoire.

[B4] World Health Organization W (2007). Global Initiative for Emergency and Essential Surgical Care (GIEESC). http://www.who.int/surgery/en/.

[B5] Debas H, Gosselin R, McCord C, Jamison D (2006). Essential Surgical Services in Africa. Disease Control Priorities in Developing Countries.

[B6] Taché S, Kaaya E, Omer S, Mkony CAE, Lyamuya E, Pallangyo K, Debas HT, MacFarlane SB (2008). University partnership to address the shortage of healthcare professionals in Africa Global Public Health. Global Public Health.

[B7] Peyre S, Peyre C, Sullivan ME, Towfigh S (2006). A surgical skills elective can improve student confidence prior to internship. J Surg Res.

[B8] Ind TE, Shelton JC, Sheperd JH (2001). Influence of training on reliability of surgical knots. BJOG.

[B9] Boehler ML, Rogers DA, Scwind CJ, Fortune J, Ketchum J, Dunnington G (2004). A senior elective designed to prepare medical students for surgical residency. American Journal of Surgery.

[B10] Kozar RA, Lucci A, Miller CC, Azizzadeh A, Cocanour  CS, Potts JR, Fischer CP, Brundage  SI (2003). Brief Intervention by surgeons can influence students toward a career in surgery. Journal of Surgery Residency.

[B11] Erzurum VZ, Obermeyer RJ, Fecher A, Azizzadeh A, Cocanour CS, Potts JR, Fischer CP, Brundage SI (2000). What influences medical students choice of surgical careers. Surgery.

[B12] Lett R (2000). Canadian Network for International Surgery: development activities and strategies. Can J Surg.

[B13] Dubrowski A, MacRae H (2006). Randomized controlled study investigating the optimal instructor: Student teacher ratios for teaching suturing skills. Medical Education.

[B14] Ethicon Inc Knot Tying Manual. http://www.medisave.co.uk/pdf/Knot_Tying_Manual.pdf.

[B15] Xu G, Hojat M (2004). A visitor's guide to effect sizes: statistical significance versus practical (clinical) importance of research findings. Advances in health sciences education.

[B16] Matsumoto ED, Hamstra SJ, Radomski SB, Cusimano MD (2002). The effect of bench model fidelity on endourological skills: a randomized controlled study. J Urol.

[B17] Naik V, Matsumoto ED, Houston P, Hamstra S, Yeung RY, Mallon JS, Martire T (2001). Fiberoptic orotracheal intubation on anesthetized, patients: Do manipulation skills learned on a simple model transfer into the operating room?. Anesthesiology.

[B18] Wanzel KR, Matsumoto ED, Hamstra SJ, Anastaskis DJ (2002). Teaching technical skills: Training on a simple, inexpensive, and portable model. Plastic and Reconstructive Surgery.

[B19] Ishumi T (2004). Tracer Studies in a Quest for Academic Improvement: Report of a study done 2002–2003.

